# External-knowledge enhanced dual encoder and contrastive learning for aspect sentiment triplet extraction

**DOI:** 10.1371/journal.pone.0340792

**Published:** 2026-03-12

**Authors:** Yuan Huang, Xiaozheng Zhou, Ruizhi Yin, Pengwei Shi

**Affiliations:** School of Information and Electrical Engineering, Hebei University of Engineering, Handan, Hebei, China; Philadelphia University, JORDAN

## Abstract

Aspect Sentiment Triplet Extraction (ASTE) is an emerging subtask of Aspect-Based Sentiment Analysis (ABSA), aiming to extract aspect terms, opinion terms, and the corresponding sentiment polarity from sentences. Many existing ASTE methods neglect to mine the deeper semantics of the sentence as well as ignore the intrinsic meanings of individual words. In order to address these limitations, this paper proposes a novel approach for ASTE. Firstly, dual encoders are used to extract the semantic and syntactic information of the sentence, the semantic encoder uses BERT and Graph Convolutional Networks (GCNs) to extract the semantic information, and the syntactic encoder employs a Bi-directional Long and Short-Term Memory (Bi-LSTM) network and GCNs to extract the syntactic information. Secondly, a feature fusion module is designed to fuse the information from the dual encoders. Finally, to enhance the ability of the model to recognize boundary tags, we design a boundary-aware contrastive learning module. Experimental results on ASTE-Data-V1 and ASTE-Data-V2 demonstrate the effectiveness of our proposed method.

## Introduction

Sentiment analysis (SA) is a crucial research area in Natural Language Processing (NLP). Traditional sentiment analysis can be categorized into document-level sentiment analysis and sentence-level sentiment analysis, but they are both coarse-grained sentiment analysis tasks and can not reflect the fine-grained sentiment information in the sentence. To address this limitation, Aspect-Based Sentiment Analysis (ABSA) [1] has emerged as a more fine-grained task in SA.

ABSA tasks are all centered around three sentiment elements, aspect terms, opinion terms and sentiment polarity. According to the different ways of combining the sentiment elements, the subtasks of ABSA can be categorized into Aspect Term Extraction (ATE) [2,3], Opinion Term Extraction (OTE) [4,5], and Aspect-Level Sentiment Classification (ASC) [6]. The objectives of ATE and OTE tasks are to extract aspect terms and opinion terms from sentences, respectively. The ASC task aims to identify the sentiment polarity of the aspect terms in sentences. However, the above tasks focus on individual sentiment elements. Based on the combination of two sentiment elements, the subtasks of ABSA can be further categorized into Aspect-Opinion pair Extraction (AOPE) [7,8], Aspect Term Extraction and Sentiment Classification (AESC) [9], AOPE is the combination of ATE and OTE tasks, while AESC is the combination of ATE and ASC tasks.

**Fig 1:**
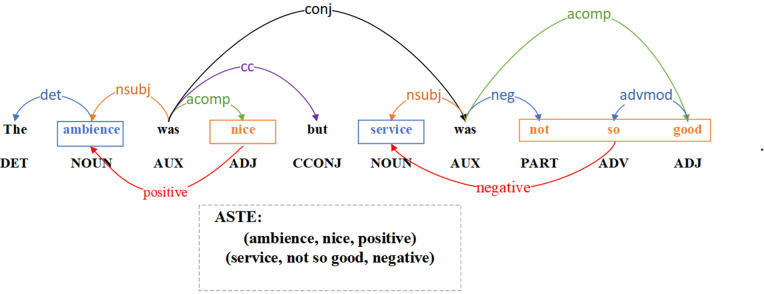
An example of ASTE.

However, all the aforementioned subtasks fail to capture the interactions between the three sentiment elements, so Peng et al. [10] proposed a novel subtask of ABSA called Aspect Sentiment Triplet Extraction (ASTE). The ASTE task aims to correctly extract the aspect terms, the associated opinion terms, and the corresponding sentiment polarity from a given sentence and to pair them accurately. Fig 1 illustrates an example of the ASTE task, for the sentence “The ambience was nice but service was not so good.,” the aspect terms are “ambience” and “service,” with their corresponding opinion terms being “nice” and “not so good” respectively. Therefore, the predicted sentiment polarity for the aspect term “ambience” is “positive” and the predicted sentiment polarity for the aspect term “service” is “negative.” The final extracted sentiment triplets from this sentence are (ambience, nice, positive) and (service, not so good, negative).

The methods of ASTE can be mainly categorized into pipeline methods and end-to-end methods. Peng et al. [10] proposed a two-stage pipeline method, which fails to consider the interactions among the three sentiment elements and is prone to error propagation. To address the drawbacks of the pipeline method, many end-to-end methods have been generated. Most of the end-to-end methods aim to explore novel tagging schemes to extract triplets. Xu et al. [11] developed an end-to-end triplet extraction framework by introducing a novel position-aware tagging scheme, achieving joint extraction for the first time. Wu et al. [12] also proposed a novel method named Grid Tagging Scheme (GTS). In addition, some researchers have also converted ASTE task into machine reading comprehension task. Chen et al. [13] converted ASTE task into a multi-round machine reading comprehension task by designing a multi-round query to recognize triplets. In addition to the above end-to-end approaches, there are other methods to extract triplets. Yan et al. [14] proposed a unified generative approach to convert the ASTE task into a generative task. Xu et al. [15] proposed a novel span-based approach, which directly captures the interactions between aspect and opinion spans. Zhang et al. [16] proposed a boundary-driven table filling method (BDTF) to extract triplets, converting the ASTE task into detection and classification of relation regions.

Although existing end-to-end methods take into account the interactions among three sentiment elements and basic semantics, they fail to fully leverage syntactic and semantic information of the sentence, often overlooking potential syntactic and semantic information in the sentence. Furthermore, these methods neglect the intrinsic information inherent in the words, which may affect the accuracy of the model for triplet extraction.

To address the above problems, this paper proposes an ASTE method that combines dual encoders with external knowledge and contrastive learning. Specifically, the proposed method integrates external knowledge of syntactic dependency tree, SenticNet and part-of-speech (POS), and dual encoders are designed to extract the higher-order syntactic and semantic information of the sentence using BERT [17], Bi-LSTM [18] and GCN [19], followed by a feature fusion mechanism to fully fuse the higher-order syntactic and semantic information, and finally based on the BDTF method to extract the triplets. In order to recognize boundary tags more accurately, we also design a boundary-aware contrastive learning module. Experimental results demonstrate that our method achieves excellent performance on the ASTE task.

The following are the contributions of this paper:

1. We propose a dual encoder approach that combines external knowledge to extract higher-order syntactic and semantic information from sentences. In addition, a feature fusion mechanism has been designed to effectively fuse the higher-order syntactic and semantic information.
2. We design a boundary-aware contrastive learning module to recognize boundary tags more accurately.
3. Experiments on the ASTE public datasets demonstrate the effectiveness of our method.

## Related work

The ASTE task encompasses three core sentiment elements: aspect terms, opinion terms, and associated sentiment polarity. Its objective is to accurately extract aspect-opinion pairs from sentences, determine their correct sentiment polarity, and properly pairing them to form sentiment triplets. Current ASTE methods can be primarily categorized into pipeline methods and end-to-end methods.

Peng et al. [10] proposed a pipeline-based two-stage approach for ASTE, where the initial stage extracts candidate aspect terms along with their corresponding sentiment polarity and candidate opinion terms, while the subsequent stage focuses on pairing the aspect terms with opinion terms. Nevertheless, this approach is prone to error propagation and fails to consider interactions among the three sentiment elements. Chen et al. [13] reformulated the ASTE task as the multi-turn machine reading comprehension (MRC) problem, where designed sequential queries effectively capture the intricate relationships among aspect terms, opinion terms, and sentiment polarity. Mao et al. [20] alternatively decomposed ASTE into dual MRC tasks, the left MRC module for aspect terms extraction and the right MRC module for extracting corresponding opinion terms and sentiment polarity given identified aspect terms. This approach implements an end-to-end framework through joint training.

While pipeline methods fail to consider interactions between sentiment elements and suffer from error propagation, Xu et al. [11] proposed a novel position-aware tagging scheme and developed an end-to-end model JET. This model is capable of jointly extracting aspect terms, opinion terms, and sentiment polarity while simultaneously capturing interactions among these three elements. Likewise, Wu et al. [12] introduced a Grid Tagging Scheme (GTS) that converts the AFOE task into a unified tagging task, eliminating error propagation issues inherent in pipeline methods. GTS offers an end-to-end approach for simultaneous multi-subtask processing. To address GTS‘s deficiencies in accurately identifying boundary detection for aspect and opinion terms, Sun et al. [21] developed an extended GTS incorporating four additional tags $\left\{A_{b},A_{i},O_{b},O_{i}\right\}$ for enhanced boundary detection.

Although novel tagging schemes have advanced in triplet extraction, most fail to model relationships between words. To address this, Chen et al. [22] proposed a multi-channel graph convolutional network to learn relation-aware node representations, transforming sentences into multi-channel graphs with words as nodes and relationships as edges. However, while GCN-based methods incorporate syntactic dependencies, they treat all dependency types equally. Yuan et al. [23] mitigated this limitation via SA-Transformer, which introduces Adjacent Edge Attention (AEA) to weight dependency types differentially, thereby more accurately capturing aspect-opinion relationships.

In addition to the end-to-end methods described above. Other methods, such as generative methods, span-based methods, table-filling methods, have emerged. Yan et al. [14] introduced a unified generative framework that reformulates ASTE as a sequence generation task, leveraging pretrained BART to extract triplets while avoiding error propagation in pipeline methods. Zhang et al. [16] developed boundary-driven table filling (BDTF) to resolve two limitations of table-filling methods: relation inconsistency and boundary insensitivity. This framework formulates the ASTE task into detection and classification of relation regions. Li et al. [24] proposed PBLUN, a novel framework for ASTE that incorporates a POS-based label update module to dynamically refine potential aspect and opinion terms through parallel execution of ATE and OTE tasks. Additionally, it enhances relation-level representations by integrating biaffine attention networks into the BDTF method. Jiang et al. [25] also proposed a method based on BDTF to solve the problem of valuable interaction information loss between aspect terms and opinion terms. Xu et al. [15] introduced a novel span-based approach that directly models the interactive relationships between aspect and opinion spans. This method generates representations for all potential aspect and opinion spans before independently predicting their associations. While this approach addresses the limitation of relying on word-level interactions for predicting the dependencies between aspect terms and opinion terms, it introduces numerous irrelevant spans that complicate model training. To mitigate this issue, Li et al. [26] proposed a span-based part-of-speech (POS) filtering method that effectively prunes irrelevant candidate spans, thereby enhancing the identification accuracy of aspect spans and opinion spans. Liu et al. [27] proposed a novel bidirectional span-based extraction framework that extracts triplets from aspect to opinion and opinion to aspect to enhance the model’s capability in processing multi-word triplets.

Although there are many methods to extract triplets, most of them do not fully mine the syntactic and semantic features of the sentence. Therefore, we propose an external knowledge enhanced dual encoder and contrastive learning extraction method to enhance the ability to extract triplets. Firstly, the semantic and syntactic features of the sentence are extracted through the semantic and syntactic encoders. Secondly, the features are fused through a feature fusion module. Finally, based on the BDTF, boundary aware contrastive learning is introduced to enhance the ability of recognizing boundary tags.

## Methodolody

**Fig 2:**
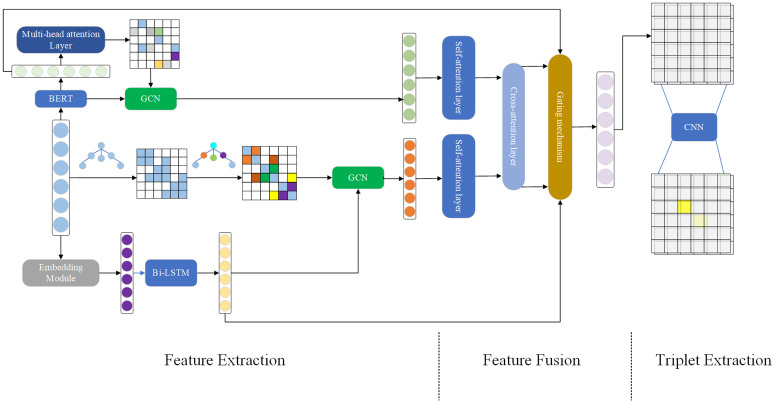
The overview of proposed method.

The proposed method consists of three major parts: feature extraction layer, feature fusion layer and triplet extraction layer. The overview of our method is shown in Fig 2. In the feature extraction layer, two encoders are designed to extract semantic and syntactic features of sentences respectively. An interaction mechanism is employed in feature fusion layer to effectively integrate features. Finally, in the triplet extraction layer, we employ the BDTF method and incorporate a boundary-aware contrastive learning module for triplet extraction.

### Task definition

Given a sentence $S=\{w_{1},w_{2},...,w_{n}\}$ containing *n* words, the objective of the ASTE task is to extract all the triplets $T=\{(a,o,p{)}_{k}{\}}_{\left(k=1\right)}^{\left|T\right|}$ of each sentence. |T| is the number of triplets and $(a,o,p{)}_{k}$ is the *k*th triplet in the sentence. a,o,p represent aspect term, opinion term, and sentiment polarity respectively. *a* and *o* consist of one or more words, and p∈{POS,NEG,NEU}.

### Feature extraction layer

#### Semantic encoder.

We first employ the pre-trained language model BERT to extract contextual semantic representation from the input sentence. Given that BERT utilizes subword tokenization, the sequence length of contextual feature representation obtained after BERT encoding may not be consistent with the original sentence length. To address this, an alignment mechanism is employed to aggregate subword-level hidden states into word-level representation, thereby maintaining dimensional consistency.


$$H^{bert}=BERT(w_{1},w_{2},...,w_{n})$$ (1)

$$H^{bert}[k]=\frac{1}{j-i}\sum_{n=i}^{j}H^{bert}[n],if\;[w_{i};w_{j}]\in w_{k}$$ (2)

where $H^{bert}[k]$ denotes the vector representation of the *k*th word after BERT coding, and $\left[w_{i};w_{j}\right]$ denotes the sequence of subwords after subword tokenization.

After acquiring the contextual semantic representation of the input sentence, the multi-head attention mechanism with relative position information is then employed to construct the attention score matrix $A^{sem}$ of the sentence. The attention scores can reflect the degree of association between words, with higher attention scores indicating a stronger association between two words, and relative position information can focus more on the neighboring words and strengthen the local dependency relationship. The process of constructing the attention score matrix as follows:


$$A_{ij}^{sem}=RMHA(H_{i}^{bert},H_{j}^{bert})$$ (3)

$$RMHA(M,N)=\frac{ContentScores+RelativeBias}{\sqrt{d/h}}$$ (4)

where *RMHA* denotes the multi-head attention mechanism with relative position information, *ContentScores* denotes the attention scores between words, *RelativeBias* denotes the bias of the relative positions of two words, and d/h denotes the dimension of each attention head.

**Fig 3:**
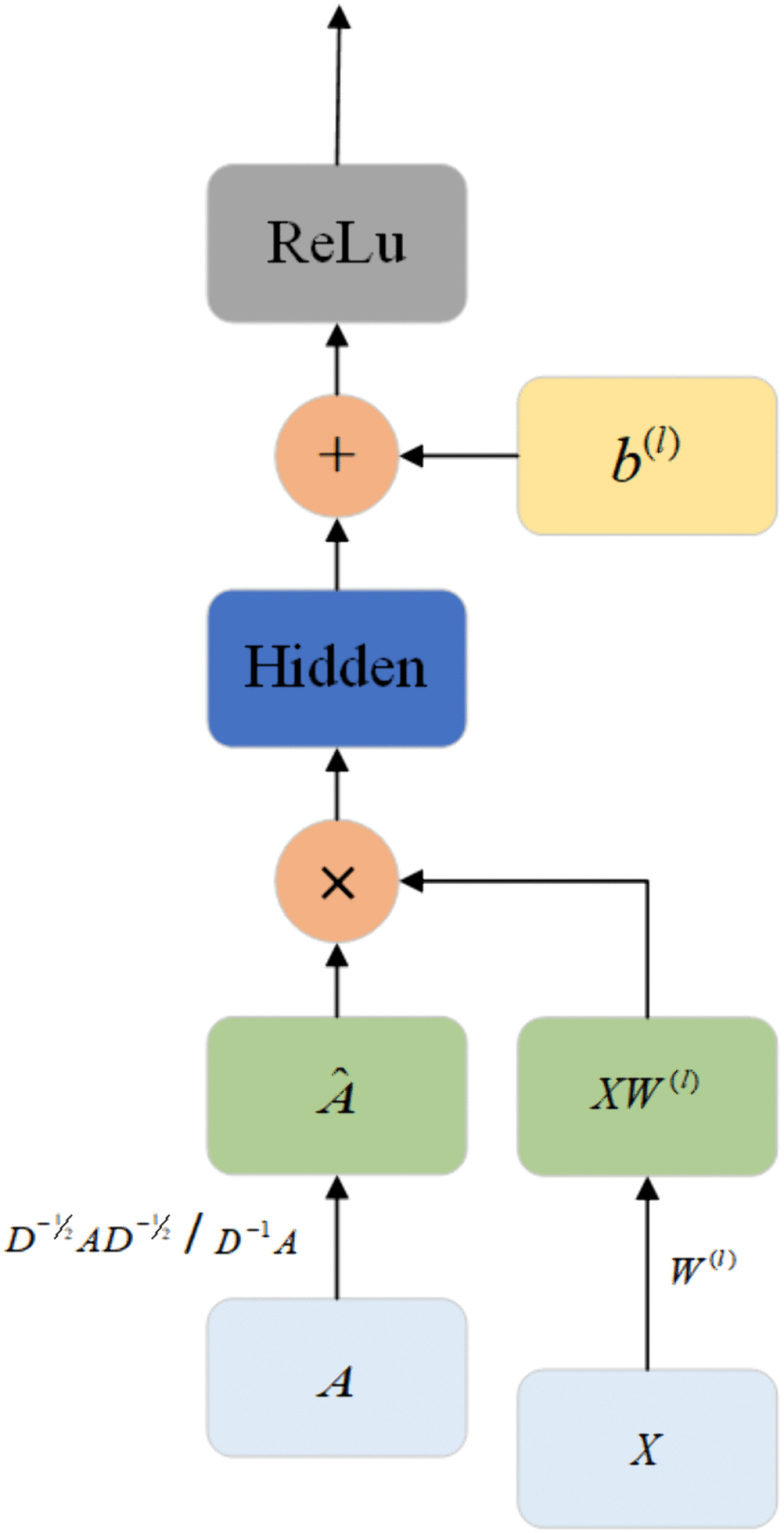
The architecture of GCN.

Finally, the attention score matrix $A^{sem}$ and the aggregated hidden state $H^{bert}$ are input into the GCN to obtain a higher-order semantic representation. Fig 3 illustrates the GCN architecture, where *A* and *X* are the adjacency matrix and input feature vector respectively, $\hat{A}$ is the normalized adjacency matrix, $W^{\left(l\right)}$ and $b^{\left(l\right)}$ are the weight matrix and bias of layer *l* respectively, Hidden represents the result of $\hat{A}XW^{\left(l\right)}$, and ReLu is the ReLU activation function. In semantic encoder, the whole process can be represented as:


$$H^{\left(k+1\right)}=\sigma (D^{-1}A^{sem}H^{\left(k\right)}W^{\left(k\right)}+b^{\left(k\right)})$$ (5)

$$D_{i,i}=\sum_{j=1}^{N}A_{i,j}^{sem}$$ (6)

where $W^{\left(k\right)}$ and $b^{\left(k\right)}$ are the weight matrix and bias of the *k*th layer respectively, $H^{\left(k\right)}$ is the representation of the *k*th layer, $H^{\left(k+1\right)}$ denotes the representation of the (*k*+1)th layer, *D* is the degree matrix, *N* denotes the number of nodes, and σ(·) denotes the activation function, and we choose ReLU function as the activation function.

The semantically enhanced contextual feature $H^{sem}$ are finally obtained after the convolution operation.

#### Syntactic encoder.

It is evident that each word possesses a richness of meaning. Within the general domain, each word has a distinct meaning, while in the special domain, the same word assumes different meanings in different contexts. Furthermore, POS plays an important role in the ASTE task as a common form of external grammatical knowledge. In the ASTE task, nouns are frequently employed as potential aspect terms, adjectives are typically considered as potential opinion terms, and other POS also plays a significant role. For instance, degree adverbs can cause sentiment polarity to become entirely opposite. In order to enhance the model‘s capacity to comprehend sentences, a fine-grained character-level embedding is introduced at the initial embedding stage. Therefore, a quadruple embedding is employed to initialize the sentence embedding, which are general domain embedding, special domain embedding, POS embedding and character-level embedding. For POS embedding, five distinct POS tags are selected, namely nouns, verbs, adjectives, adverbs and others to construct a POS embedding table. This table is initialized randomly and subsequently updated during the training process. For character-level embedding, CNN is utilized to obtain character-level embedding for each word. Therefore, the final initial embeddings $E\in {\mathbb{R}}^{n\times (d_{g}+d_{s}+d_{p}+d_{c})}$ are as follows:


$$E=E_{g}⊕E_{s}⊕E_{p}⊕E_{c}$$ (7)

where $E_{g}\in {\mathbb{R}}^{\left(n\times d_{g}\right)}$, $E_{s}\in {\mathbb{R}}^{\left(n\times d_{s}\right)}$, $E_{p}\in {\mathbb{R}}^{\left(n\times d_{p}\right)}$, and $E_{c}\in {\mathbb{R}}^{\left(n\times d_{c}\right)}$ denote the general domain embedding table, special domain embedding table, POS embedding table and character-level embedding table, respectively.

After obtaining the initial embeddings, the Bi-LSTM is employed to extract the contextual feature of the input sentence. The Bi-LSTM processes the input sequence in both forward and backward directions. The final result, $H^{bilstm}\in R^{\left(n\times d_{l}\right)}$, is obtained by concatenating the results from the forward and backward directions.


$$\vec{H^{b}}=\vec{LSTM}(E)$$ (8)

$$\overset{\leftarrow }{H^{b}}=\overset{\leftarrow }{LSTM}(E)$$ (9)

$$H^{bilstm}=\vec{H^{b}}⊕\overset{\leftarrow }{H^{b}}$$ (10)

where $d_{l}$ denotes the dimension of the hidden state of the Bi-LSTM, ⊕ indicates the concatenation operation.

In order to obtain higher-order syntactic representation, two types of external knowledge are introduced: SenticNet [28] and the syntactic dependency tree. A external knowledge-enhanced adjacency matrix is constructed and modeled using GCNs to capture and enhance the syntactic structure of the sentence. The syntactic dependency tree shows the dependencies between words. In the ASTE task, syntactic dependency analysis can facilitate the identification of the relationship between aspect term and opinion term. As illustrated in Fig 1, the dependencies between words are depicted in a syntactic dependency tree. In SenticNet, sentiment scores are employed to indicate the polarity and intensity of each word, each word possesses a sentiment score ranging from −1–1. Sentiment score that approach −1 for a word signifies that the word is more negative, sentiment score that approach 1 indicates that the word is more positive, and sentiment score of 0 for a word denotes that the word possesses a neutral sentiment polarity or indicates the absence of the word from the SenticNet. The sentiment information of aspect and opinion terms can be enhanced by introducing the SenticNet. An example of SenticNet is shown in Table 1.

**Table 1:** An example of SenticNet.

word	SenticNet(word)
excellent	0.744
hard	-0.81
good	0.849
cramped	-0.68
food	0.054

To fully leverage the syntactic information of the sentence, the construction of an external knowledge-enhanced adjacency matrix is required. Specifically, a syntactic dependency matrix $T\in {\mathbb{R}}^{n\times n}$ is generated through syntactic dependency parsing. The construction rule is as follows: if a dependency relationship exists between two words, the corresponding position in the matrix is set to 1, as well as the diagonal elements. Conversely, if no relationship exists, the corresponding position in the matrix is set to 0. The construction procedure is as follows:


$$\begin{array}{c} T_{ij}=\left\{ \begin{array}{cc} 1 & \text{if }w_{i},w_{j}\text{ contains dependency}\\ 0 & \text{otherwise} \end{array}\right.\; \end{array}$$ (11)

Following the acquisition of the syntactic dependency matrix, the sentiment intensity adjacency matrix $Z\in {\mathbb{R}}^{n\times n}$ is then constructed, and the sentiment information between two words is computed through summation of their individual sentiment scores. The construction rule for the sentiment intensity adjacency matrix is as follows:


$$Z_{ij}=SenticNet(w_{i})+SenticNet(w_{j})$$ (12)

The final external knowledge-enhanced adjacency matrix $A^{syn}$ is derived through integration of the syntactic dependency matrix T with the sentiment intensity adjacency matrix Z.


$$A_{ij}^{syn}=T_{ij}\times (Z_{ij}+1)$$ (13)

Finally, external knowledge-enhanced adjacency matrix $A^{syn}$ and the contextual representation $H^{bilstm}$ derived from the Bi-LSTM are input into the GCN to obtain a higher-order syntactic representation, and the whole process can be represented as:


$$H^{\left(k+1\right)}=\sigma (D^{-1/2}A^{syn}D^{-1/2}H^{\left(k\right)}W^{\left(k\right)}+b^{\left(k\right)})$$ (14)

$$D_{i,i}=\sum_{j=1}^{N}A_{i,j}^{syn}$$ (15)

where $W^{\left(k\right)}$ and $b^{\left(k\right)}$ are the weight matrix and bias of the kth layer respectively, $H^{\left(k\right)}$ is the representation of the *k*th layer, $H^{\left(k+1\right)}$ denotes the representation of the (*k*+1)th layer, *D* is the degree matrix, *N* denotes the number of nodes, and σ(·) denotes the activation function, and we choose ReLU function as the activation function.

The syntactically enhanced contextual feature $H^{syn}$ are finally obtained after the convolution operation.

### Feature fusion layer

Following the acquisition of syntactically enhanced contextual feature and semantically enhanced contextual feature, an interaction mechanism for feature fusion is designed. Firstly, a self-attention mechanism is applied to the syntactically enhanced contextual feature and the semantically enhanced contextual feature, respectively, in effects to more effectively capture the dependencies between the features. Subsequently, the cross-attention mechanism is employed to derive the syntax-enhanced semantic feature $H^{esem}$ and the semantics-enhanced syntactic feature $H^{esyn}$.

The self-attention mechanism is formulated as:


$$SA(X)=softmax(\frac{(XW^{Q})(XW^{K}{)}^{T}}{\sqrt{d_{k}}})(XW^{V})$$ (16)

where X denotes the input sequence, $W^{Q},W^{K},W^{V}$ denote the weight matrixs, and $d_{k}$ denotes the dimension of the hidden state, respectively.

Therefore, self-attention operations are performed on $H^{sem}$ and $H^{syn}$, respectively:


$$H^{sa-sem}=SA(H^{sem})$$ (17)

$$H^{sa-syn}=SA(H^{syn})$$ (18)

The cross-attention mechanism is formulated as:


$$CA(X,Y)=softmax(\frac{(XW^{Q})(YW^{K}{)}^{T}}{\sqrt{d_{k}}})(YW^{V})$$ (19)

where *X*,*Y* denote the input sequences, $W^{Q},W^{K},W^{V}$ denote the weight matrixs, and $d_{k}$ denotes the dimension of the hidden state, respectively.

Therefore, cross-attention operations are performed on $H^{sa-sem}$ and $H^{sa-syn}$, respectively:


$$H^{esem}=CA(H^{sa-sem},H^{sa-syn})$$ (20)

$$H^{esyn}=CA(H^{sa-syn},H^{sa-sem})$$ (21)

Consequently, in order to prevent the loss of critical features, we consider the initially obtained semantic feature $H^{bert}$ and the syntactic feature $H^{bilstm}$ and design a gating mechanism to fuse the four features to obtain the final feature representation $H^{final}$.


$$H^{final}=\sum_{i=1}^{4}\frac{\exp(W_{i}x_{i}+b_{i})}{\sum_{j=1}^{4}\exp(W_{j}x_{j}+b_{j})}x_{i}$$ (22)

where $x_{1},x_{2},x_{3},x_{4}$ denote $H^{esem},H^{esyn},H^{bert}$ and $H^{bilstm}$ respectively.

The final fused vector is represented as:


$$H^{final}=\{{\tilde{h}}_{1},{\tilde{h}}_{2},{\tilde{h}}_{3},...,{\tilde{h}}_{n}\}$$ (23)

### Triplet extraction layer

**Fig 4:**
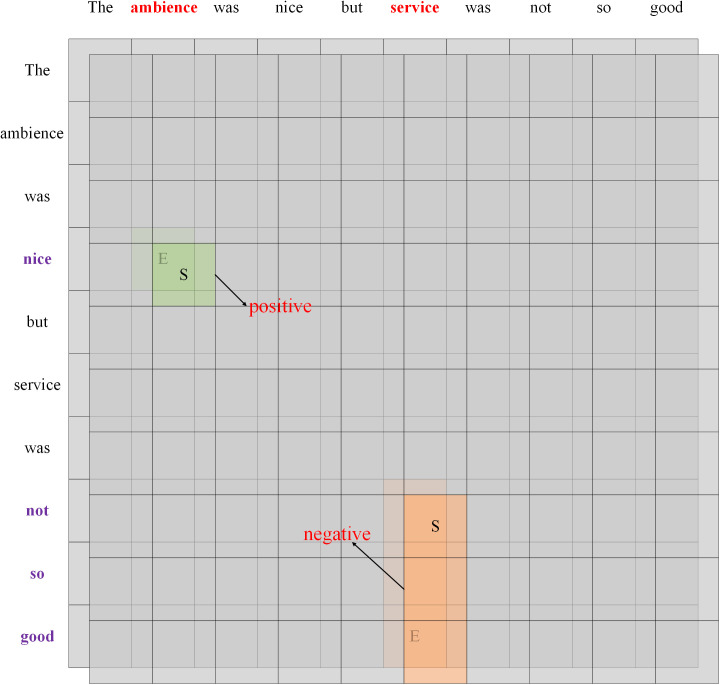
An example of BDTF.

The BDTF [10] method is utilized for the extraction of triplets. A distinguishing feature of the BDTF method is its construction of a table, identification of the regions by S and E tags, and subsequent application of sentiment judgement to the regions. This approach diverges from other table-filling methods. As illustrated in Fig 4, an example of BDTF is presented. The S tag denotes the intersection point between the starting positions of the aspect term and the opinion term, while the E tag represents the intersection point between their ending positions. The region determined by the S tag and E tag constitutes the relationship region.

Firstly, it is necessary to construct the relation-level representation. The final feature representation, $H^{final}$, is used to construct the relation-level representation. Contextual information between words is also significant, which is obtained by means of a maximum pooling operation. The representations of the two words of the word pair, the contextual representation, and the interaction representation of the two words are concatenated. The concatenated representations are then passed through a nonlinear projection and a gelu activation function [29] to obtain the final relation-level representation. The representation of each word pair can be expressed by the following equation:


$$r_{ij}=gelu(Linear([\tilde{h_{i}},\tilde{h_{j}},c_{ij},t_{ij}]))$$ (24)

$$c_{ij}=pooling([\tilde{h_{i}},\tilde{h_{i+1}},...,\tilde{h_{j}}])$$ (25)

$$t_{ij}=h_{i}^{T}Vh_{j}$$ (26)

where $c_{ij}$ denotes the maximum pooling operation, $t_{ij}$ denotes the interaction representation between two words, and $V\in {\mathbb{R}}^{d\times d\times t}$ is the tensor parameter.

Following the acquisition of the relation-level representation of each word pair, the relation-level representations of all word pairs are assembled to form a 3D relation table $R\in {\mathbb{R}}^{n\times n\times d}$. There may be some potential dependencies between relation-level representations of word pairs, and to extract such dependencies, the *L* layer ResNet-style CNN [30] is adopted. The table representation obtained from the *l* layer CNN is denoted as $T^{\left(l\right)}$:


$$T^{\prime }=ReLU(LN(Conv_{1\times 1}(T^{\left(l-1\right)})))$$ (27)

$$T^{\prime \prime }=ReLU(LN(Conv_{3\times 3}(T^{\prime })))$$ (28)

$$T^{\prime \prime \prime }=ReLU(LN(Conv_{1\times 1}(T^{\prime \prime })))$$ (29)

$$T^{\left(l\right)}=T^{\prime \prime \prime }+T^{\left(l-1\right)}$$ (30)

where LN denotes Layer Normalization [31] and the initial input table $T^{\left(0\right)}=R$.

The final table representation, $T^{\left(L\right)}$, is obtained and subsequently two biaffine attention networks [24] are used to enhance the representations of S and E tags. In order to prevent overfitting, two multi-layer perceptrons are employed.


$$h_{i}={MLP}_{1}(\tilde{h_{i}})$$ (31)

$$h_{j}={MLP}_{2}(\tilde{h_{j}})$$ (32)

The formula of biaffine attention network as follows:


$$s_{i,j}^{x_{i}}=h_{i}^{T}U_{1}^{x_{i}}h_{j}+U_{2}^{x_{i}}(h_{i}⊕h_{j})+b^{x_{i}}$$ (33)

where $U_{1}^{x_{i}},U_{2}^{x_{i}}$ and $b^{x_{i}}$ are trainable weights and biases, we can obtain $s_{i,j}^{S}$ and $s_{i,j}^{E}$ by performing biaffine operation on S and E tags, respectively, and then we can obtain the probability scores by performing sigmoid operation on $s_{i,j}^{S}$ and $s_{i,j}^{E}$:


$$P_{i,j}^{B-S}=sigmoid(s_{i,j}^{S})$$ (34)

$$P_{i,j}^{B-E}=sigmoid(s_{i,j}^{E})$$ (35)

Subsequently, the probability scores obtained after the biaffine attention network are utilized to enhance the representations of the original S and E tags. Following the acquisition of the enhanced representations, two classifiers are employed to determine the candidate S and E tags. Thereafter, the top-*k* strategy is implemented to prune the candidate S and E tags, resulting in the identification of the final boundary S and E tags.


$$P_{ij}^{S}=top-k(sigmoid((1+P_{i,j}^{B-S})⊗Linear(r_{ij}^{\left(L\right)})))$$ (36)

$$P_{ij}^{E}=top-k(sigmoid((1+P_{i,j}^{B-E})⊗Linear(r_{ij}^{\left(L\right)})))$$ (37)

**Fig 5:**
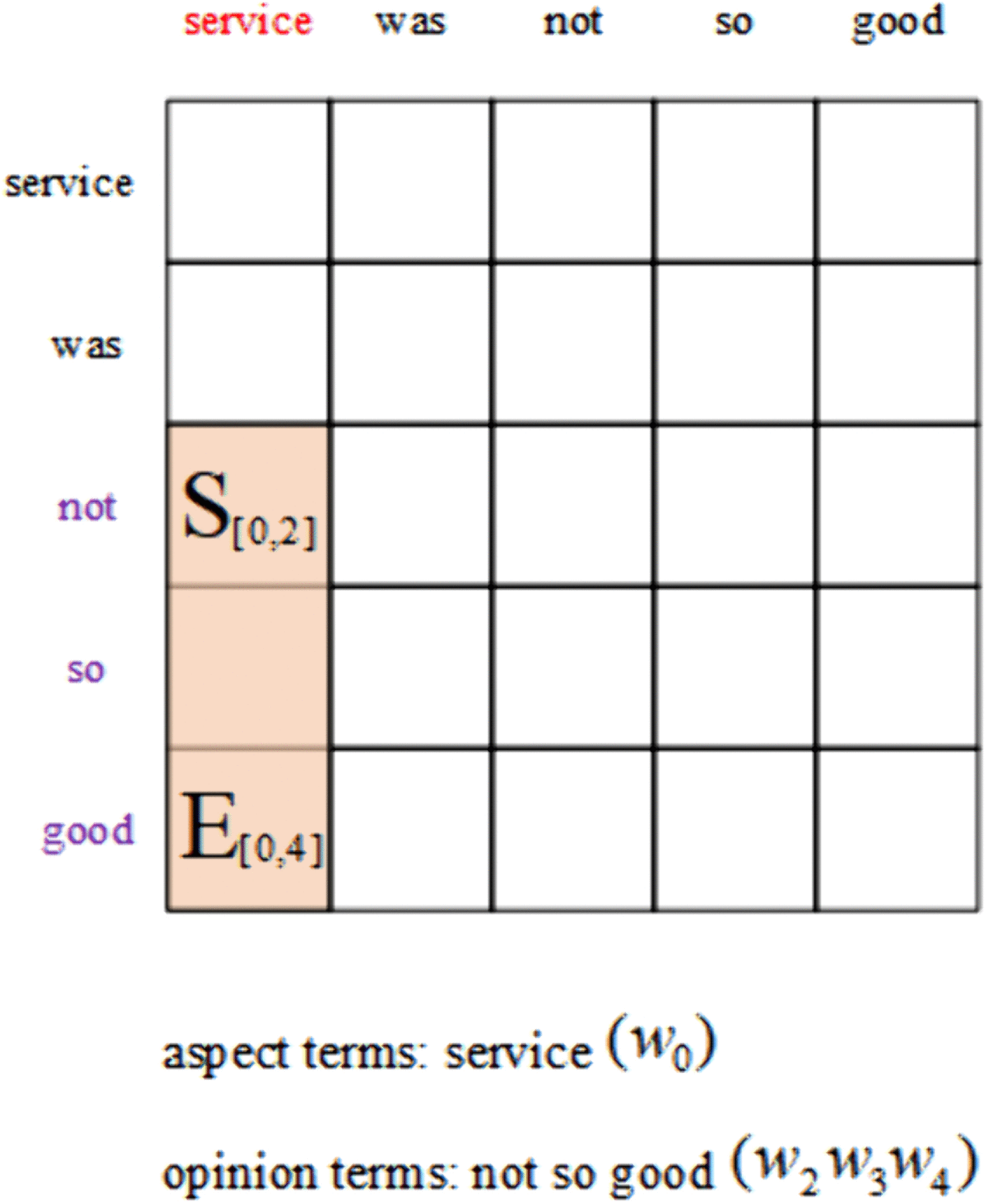
Correspondence between S tags, E tags, aspect terms, and opinion terms.

With the final boundary S and E tags, the relationship regions have been determined. Given $S_{\left[a,b\right]}$ and $E_{\left[c,d\right]}$, if a,b,c,d satisfy a≤c,b≤d,the relation region is a valid region. Once the relationship region is determined to be a valid region, then aspect terms and opinion terms can be extracted, since there exists the aforementioned correspondence a between S tags, E tags, aspect terms, and opinion terms. For sentence $S=\{w_{1},w_{2},...,w_{a},...,w_{c},...,w_{b},...,w_{d},...,w_{n}\}$, where aspect terms are $\left(w_{a},...,w_{c}\right)$ and opinion terms are $\left(w_{b},...,w_{d}\right)$. Fig 5 demonstrates an example of aspect term and opinion term extraction.For each relation region, the representation of the S tag, the representation of the E tag, and the maximal pooling result of the relation region are used to construct a representation of the relation region. Finally, a classifier is employed to predict the sentiment polarity of the relation region:


$$r_{abcd}=[r_{ab}^{\left(L\right)},r_{cd}^{\left(L\right)},p_{abcd}^{\left(L\right)}]$$ (38)

$$\begin{array}{c} p_{abcd}^{\left(L\right)}=pooling(\begin{array}{ccc} r_{ab}^{\left(L\right)} & \cdots & r_{ad}^{\left(L\right)}\\ \vdots & \ddots & \vdots \\ r_{cb}^{\left(L\right)} & \cdots & r_{cd}^{\left(L\right)} \end{array})\; \end{array}$$ (39)

$$P_{abcd}({𝒴}_{p})=softmax(Linear(r_{abcd}))$$ (40)

where ${𝒴}_{p}\in \{POS,NEG,NEU\}$.

### Boundary-aware contrastive learning

In ASTE task, it is imperative to accurately identify S and E tags. Aspect terms, opinion terms and relationship regions are determined by S and E tags. To facilitate the model‘s ability to identify S and E tags, a boundary-aware contrastive learning module has been developed. This module employs contrastive learning on both S and E tags to optimize their respective representations, thereby enhancing the model‘s recognition capability.

Initially, each word pair representation in the complete table representation is represented as vector representations. After processing through the ResNet CNN, the complete table representation is obtained, which consequently the representation for each individual word pair has also been obtained. The table representation is denoted as table, thus the representation of each word pair is denoted as $table[x_{0},x_{1}]$.

After obtaining the representation of each word pair, the next is to construct positive and negative samples. For multi-word aspect terms and multi-word opinion terms, the model may bias the identification of S and E tags, resulting in inaccurate identification of the start and end positions of aspect terms and opinion terms. To address this, a methodology is employed that involves the selection of positive and negative samples. Specifically, the correct S tags within the same triplet are designated as positive samples, while the surrounding positions of the correct S tags are utilized as negative samples.The correct S tag $S_{\left[a,b\right]}$ within the same triplet is first retrieved and represented as table[a,b], and $S_{\left[a,b\right]}^{i}$ and $table[a,b{]}^{i}$ represent the correct S tag and S tag representation of the *i*th triplet, respectively. By superimposing Gaussian random noise on the correct S tag representation, the positive sample representation $h_{pos}^{i}$ and anchor sample representation $h_{anchor}^{i}$ are obtained, where the noise follows a normal distribution with a mean of 0 and a standard deviation of 0.1. This process can be formally expressed as:


$$h_{pos}^{i}=table[a,b{]}^{i}+noise$$ (41)

$$h_{anchor}^{i}=table[a,b{]}^{i}+noise$$ (42)

The surrounding positions of correct S tag $S_{\left[a,b\right]}$ are selected as negative samples, and negative sample representations can be represented as table[a,b−1], table[a,b+1], table[a−1,b], table[a+1,b]. For the *i*th triplet, its negative sample representation is denoted as $h_{neg}^{i,j}=\{table[a,b{]}^{i,1},table[a,b{]}^{i,2},...,table[a,b{]}^{i,j}\}$.

Subsequent to the construction of the positive and negative samples, the contrastive learning loss function is utilized to calculate the distance between the positive and negative samples and to update the model parameters. In our approach, Triplet Contrastive Loss is employed to minimize the distance between anchor samples and positive samples while maximizing the distance between anchor samples and negative samples, thereby learning discriminative feature representations. Thus, the contrastive loss for the S tag of the *i*th triplet is:


$${ℒ}_{S}^{i}=\frac{1}{N}\sum_{j=1}^{N}\max(0,{\left\|h_{anchor}^{i}-h_{pos}^{i)}\right\|}_{2}-{\left\|h_{anchor}^{i}-h_{neg}^{i,j}\right\|}_{2}+margin)$$ (43)

where *N* denotes the number of samples of the *i*th triplet, $h_{neg}^{i,j}$ denotes the vector representation of the *j*th negative sample in the *i*th triplet.

Therefore, he contrastive loss for S tags can thus be expressed as:


$${ℒ}_{S}=\sum_{k=1}^{M}{ℒ}_{S}^{i}$$ (44)

where *M* denotes the number of triplets.

Similarly, the contrastive loss ${ℒ}_{E}$ can be obtained for the E tags.

### Training loss

The final loss ℒ consists of three components, the loss of boundary detection ${ℒ}_{B}$, the loss of region classification ${ℒ}_{p}$ and the loss of contrastive learning ${ℒ}_{cl}$.


$$ℒ={ℒ}_{B}+{ℒ}_{p}+{ℒ}_{cl}$$ (45)

For the loss of boundary detection ${ℒ}_{B}$:


$${ℒ}_{B}={ℒ}_{B-S}+{ℒ}_{B-E}$$ (46)

$${ℒ}_{B-S}=-\sum_{i,j\in [1,n]}{𝒴}_{ij}^{S}\log P_{ij}^{S}+(1-{𝒴}_{ij}^{S})\log\left(1-P_{ij}^{S}\right)$$ (47)

$${ℒ}_{B-E}=-\sum_{i,j\in [1,n]}{𝒴}_{ij}^{E}\log P_{ij}^{E}+(1-{𝒴}_{ij}^{E})\log\left(1-P_{ij}^{E}\right)$$ (48)

where ${𝒴}_{ij}^{S}$ and ${𝒴}_{ij}^{E}$ are the true boundary tags, $P_{ij}^{S}$ and $P_{ij}^{E}$ are the predicted boundary tags.

For the loss of region classification ${ℒ}_{p}$:


$${ℒ}_{p}=-\sum_{a,b,c,d\in C_{R}}\log\left(P_{abcd}({𝒴}_{p}^{*})\right)$$ (49)

where ${𝒴}_{p}^{*}$ is the true region type.

For the loss of contrastive learning ${ℒ}_{cl}$:


$${ℒ}_{cl}={ℒ}_{S}+{ℒ}_{E}$$ (50)

where ${ℒ}_{S}$ and ${ℒ}_{E}$ denote the contrastive loss of the S tags and the contrastive loss of the E tags, respectively.

## Experiments

### Datasets

We conducted comprehensive evaluations of our proposed method on two benchmark datasets for ASTE task: ASTE-Data-V1 [10] and ASTE-Data-V2 [11]. Both datasets comprise four sub-datasets with distinct domains: three restaurant review datasets (14res, 15res, and 16res) and one laptop review dataset (14lap). Table 2 presents the detailed statistics of these datasets.

**Table 2:** The detailed statistics of ASTE-Data-V1 and ASTE-Data-V2 datasets.

Dataset		14lap			14res			15res			16res		
		Train	Dev	Test	Train	Dev	Test	Train	Dev	Test	Train	Dev	Test
V1	#S	920	228	339	1300	323	496	593	148	318	842	210	320
	#A	1283	317	475	2079	530	849	834	225	426	1183	291	444
	#O	1265	337	490	2145	524	862	923	238	455	1289	316	465
	#T	1265	337	490	2145	524	862	923	238	455	1289	316	465
V2	#S	906	219	328	1266	310	492	605	148	322	857	210	326
	#A	1280	295	463	2051	500	848	862	213	432	1198	296	452
	#O	1254	302	466	2061	497	844	935	236	460	1300	319	474
	#T	1460	346	543	2338	577	994	1013	249	485	1394	339	514

#S, #A, #O, #T denote the number of sentences, aspect terms, opinion terms and triplets respectively.

### Experimental settings

In the semantic encoding module, “bert-base-uncased” is employed as the encoder, and the layers of GCN are two layers. ReLU function is used in GCN architecture. The feature dimensions for both input and output representations of each GCN layer are 768. And random-walk normalization is employed to preprocess the adjacency matrix. In the syntactic encoding module, Spacy is utilized for syntactic dependency parsing and NLTK is employed for POS tagging. The hidden layer dimension of the Bi-LSTM is 300. In the word embedding module, we use a 100-dimensional 1D convolutional kernel for CNN character embedding and 300-dimensional pre-trained word vectors from Glove [32] for general domain word embedding. We also use 500-dimensional word embeddings from the Amazon Product Review Corpus [33] for special domain word embedding and 100-dimensional random initial vectors for POS embeddings. The GCN uses two layers. The feature dimensions for both input and output representations of each GCN layer are 600. And symmetrically normalization is employed to preprocess the adjacency matrix. In GCN, both weight matrices and bias terms are initialized by sampling from a uniform distribution. The number of layers is set to 1 for both MLPs, each with an input dimension of 768 and an output dimension of 300. ReLU fuction is employed in MLPs. The version of SenticNet is SenticNet 6. AdamW [34] is used as the optimizer. The model is trained for 15 epochs using different random seeds, and the model that performs best on the validation set is selected to test on the testing set.

### Baselines

In order to demonstrate the effectiveness of our proposed method, we compared it with several state-of-art ASTE methods. These ASTE methods can be divided into three categories: pipeline-based methods, span-based methods and joint extraction methods.

**Pipeline-based methods**

- **Peng-two-stage:** [10] proposed a two-stage framework for the ASTE. In the initial stage, candidate aspect terms, along with their corresponding sentiment polarity and candidate opinion terms are extracted. In the next stage, aspect terms, sentiment polarity and opinion terms are paired.

**Span-based methods**

- **Span-ASTE:** [15] was the first to explicitly model interactions between aspect spans and opinion spans in order to address sentiment inconsistent of word pair interactions.
- **Literature [27]:** [27] proposed a framework for bidirectional extraction that was based on spans. The design of two decoders from aspect to opinion and from opinion to aspect was achieved, and bi-directional extraction was realized through the modelling of the relationship between spans by means of a multi-head attention mechanism.

**Joint extraction methods**

- **JET:** [11] proposed an innovative position-aware tagging scheme for joint sentiment triplet extraction. The method is capable of more effective capture of interactions among sentiment elements.
- **GTS:** [12] proposed a grid tagging scheme and developed an inference strategy to explore the relationships between different opinion factors.
- **EMC-GCN:** [22] employed multi-channel graphs to capture dependency relationships between words and enhanced the model with linguistic features.
- **BDTF:** [16] employed relation regions in a 2D table to represent sentiment triplets, thereby converting the ASTE task into a detection and classification task for relation regions.
- **SA-Transformer:** [23] incorporated dependency types into graph neural networks and proposed AEA, which learns different representations and weights for each edge through dependency types.
- **Literature [21]:** [21] proposed a novel grid tagging scheme, representing an extension of the GTS. The addition of four tags, $A_{b},A_{i},O_{b},O_{i}$, to GTS is intended to enhance the boundary recognition of aspect and opinion terms.
- **PBLUN:** [24] proposed a POS-based label update module to provide more accurate tags of aspect terms and opinion terms for subsequent tasks, and utilized two biaffine attention networks to enhance the representation of S and E tags in the BDTF method.

### Experimental results

As demonstrated in Table 3, the experimental results of our method and other benchmark models on the four datasets of ASTE-Data-V2 are presented. Similarly, Table 4 shows the experimental results of our method and other benchmark models on the four datasets of ASTE-Data-V1. The evaluation of the models employs three evaluation metrics: F1 score (F1), Precision (P) and Recall (R). The experimental results demonstrate the efficacy of the proposed method on all datasets.

**Table 3:** Experimental results on ASTE-Data-V2.

	Model	14lap			14res			15res			16res		
		P	R	F1	P	R	F1	P	R	F1	P	R	F1
Pipeline-based methods	Peng-two-stage	37.38	50.38	42.87	43.24	63.66	51.46	48.07	57.51	52.32	46.96	64.24	54.21
Span-based methods	Span-ASTE	63.44	55.84	59.38	72.89	70.89	71.85	62.18	64.45	63.27	69.45	71.17	70.26
	Literature[27]	66.81	56.98	61.51	74.88	70.64	72.70	65.93	63.07	64.47	70.36	72.29	71.31
Joint extraction methods	JET(M=6)-BERT	55.39	47.33	51.04	70.56	55.94	62.40	64.45	51.96	57.53	70.42	58.37	63.83
	GTS-BERT	57.82	51.32	54.36	67.76	67.29	67.50	62.59	57.94	60.15	66.08	66.91	67.93
	EMC-GCN	61.70	56.26	58.81	71.21	72.39	71.78	61.54	62.47	61.93	65.62	71.30	68.33
	BDTF	68.94	55.97	61.74	75.53	73.24	74.35	68.76	63.71	66.12	71.44	73.13	72.27
	SA-Transformer	61.28	48.98	54.44	70.76	65.85	68.22	62.82	58.31	60.48	72.01	62.87	67.13
	Literature[21]	58.97	46.77	52.17	70.70	63.07	66.67	65.08	50.72	57.01	69.91	60.23	64.71
	PBLUN	66.84	**58.71**	62.51	**76.97**	73.20	**75.03**	69.64	64.33	66.88	72.11	73.74	72.89
	**Ours**	**70.16**	58.23	**63.64**	76.60	**73.44**	74.99	**72.12**	**64.54**	**68.12**	**74.07**	**73.93**	**74.00**

The experimental results for the baselines are taken from published papers, with the best experimental results in bold and the second best underlined.

**Table 4:** Experimental results on ASTE-Data-V1.

	Model	14lap			14res			15res			16res		
		P	R	F1	P	R	F1	P	R	F1	P	R	F1
Pipeline-based methods	Peng-two-stage	40.40	47.24	43.50	44.18	62.99	51.89	40.97	54.68	46.79	46.76	62.97	53.62
Joint extraction methods	JET(M = 6)-BERT	58.47	43.67	50.00	67.97	60.32	63.92	58.35	51.43	54.67	64.77	61.29	62.98
	GTS-BERT	57.52	51.92	54.58	70.92	69.49	70.20	59.29	58.07	58.67	68.58	66.60	67.58
	EMC-GCN	61.46	55.56	58.32	71.85	72.12	71.98	59.89	61.05	60.38	65.08	71.66	68.18
	BDTF	68.30	55.10	60.99	76.71	74.01	75.33	66.95	**65.05**	65.97	73.43	73.64	73.51
	Literature [21]	61.82	46.06	52.79	72.96	62.85	67.53	66.23	50.91	57.57	74.12	64.05	68.72
	**Ours**	**69.14**	**57.14**	**62.57**	**77.38**	**74.59**	**75.96**	**70.98**	**65.05**	**67.89**	**76.39**	**73.76**	**75.05**

The experimental results for the baselines are taken from published papers, with the best experimental results in bold and the second best underlined.

As presented in Table 3, compared to the Peng-two-stage model, our proposed method achieves a 20.77%,23.53%,15.8%, and 19.79% enhancement in the F1 score metrics, respectively. In comparison with the latest span-based method [27], our method enhances the F1 scores by 2.13%,2.29%,3.65%, and 2.69%, respectively. When evaluated against the BDTF, our method shows enhancements of 1.9%, 0.64%, 2%, 1.73% on four datasets. In comparison with the PBLUN framework, our method demonstrates improvements of 1.13%,1.24%, and 1.11% on the 14lap, 15res, and 16res datasets respectively.

As shown in Table 4, compared with the Peng-two-stage model, our method achieves F1 score improvements of 19.07%,24.07%,21.10%,21.43% on the four datasets, respectively. Relative to the table-filling based method BDTF, our method achieves improvements of 1.58%, 0.63%, 1.92%, 1.54% in F1 score.

The experimental results demonstrate the efficacy of the proposed method, substantiating its superior capacity to extract the deep syntactic and semantic information of sentences. Furthermore, it is evident that the efficacy of boundary-aware contrastive learning module in our approach is effective in enhancing the representation of S and E tags.

### Ablation study

**Fig 6:**
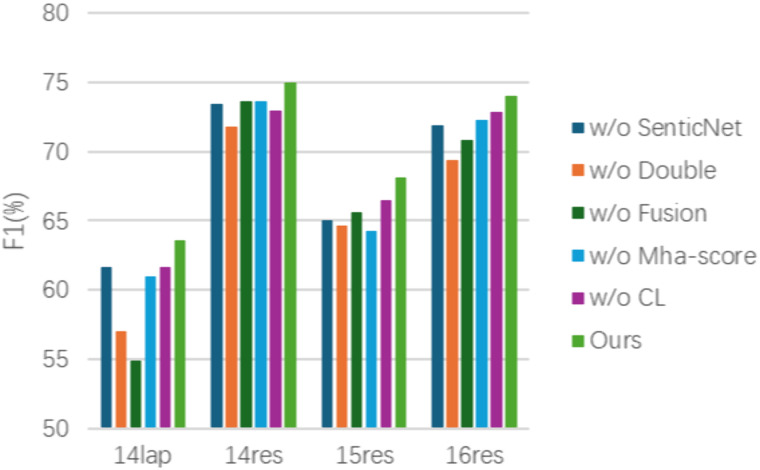
The experimental results of ablation study on ASTE-Data-V2.

In order to evaluate the validity of the different modules in our method, we conducted an ablation study on four datasets from ASTE-Data-V2, and chose the F1 score as the evaluation metric. As shown in Table 5 and Fig 6, the experimental results of the ablation study are presented. “w/o SenticNet” indicates that no SenticNet is utilized to enhance the adjacency matrix, “w/o Double” indicates that only a single encoder is employed, and “w/o Fusion” indicates that the feature fusion module is not used and only the features are simply added. “w/o MHA-score” denotes the absence of the construction of an attention score matrix with relative position information, with the results of the BERT encoder being utilized directly instead. “w/o CL” indicates the absence of the use of boundary-aware contrastive learning module to enhance the representations of S and E tags.

**Table 5:** The results of ablation study on ASTE-Data-V2.

Model	14lap	14res	15res	16res
w/o SenticNet	61.66(−1.98)	73.45(−1.54)	65.09(−3.03)	71.91(−2.09)
w/o Double	57.06(−6.58)	71.83(−3.16)	64.68(−3.44)	69.36(−4.64)
w/o Fusion	54.95(−8.69)	73.65(−1.34)	65.61(−2.51)	70.87(−3.13)
w/o Mha-score	61.03(−2.61)	73.66(−1.33)	64.25(−3.87)	72.26(−1.74)
w/o CL	61.66(−1.98)	73.00(−1.99)	66.53(−1.59)	72.87(−1.13)
Ours	63.64	74.99	68.12	74.00

In comparison to our proposed method, employing the BERT single encoder for encoding results in a significant decline in model performance, with a decrease in F1 scores by 6.58%,3.16%,3.44%, and 4.64% on the four datasets, respectively. The construction of the adjacency matrix without the utilization of the SenticNet resulted in a decline in the F1 scores across the four datasets, with a decrease of 1.98%, 1.54%, 3.03%, and 2.09%, respectively. This demonstrates that incorporating the sentiment information of individual words enhances the extraction of sentiment triplets. Without the feature fusion mechanism, simply features addition results in the loss of valuable features and consequently leads to inaccuracy in extracting sentiment triplets. The employment of features addition leads to a decline in F1 scores by 8.69%, 1.34%, 2.51% and 3.13%.

The study of the boundary-aware contrastive learning approach demonstrates that the absence of the boundary-aware contrastive learning module results in a decline in F1 scores on the four datasets, with a decrease of 1.98%, 1.99%, 1.59%, and 1.13%, respectively. The incorporation of the boundary-aware contrastive learning module facilitates the model‘s capacity to accurately identify boundary S and E tags, thereby enhancing the effect of triplets extraction.

The investigation into the attention score matrix reveals that not constructing the attention score matrix with relative position information leads to a decline in F1 scores by 2.61%, 1.33%, 3.87%, and 1.74%, respectively, when compared with our method on the four datasets. This suggests that the attention score matrix with relative position information reflects the different importance of the interactions between the words and makes the model more capable of identifying pairs of words with stronger relations, thereby facilitating the recognition of aspect terms and opinion terms.

### Effect of the number of GCN layers

**Fig 7:**
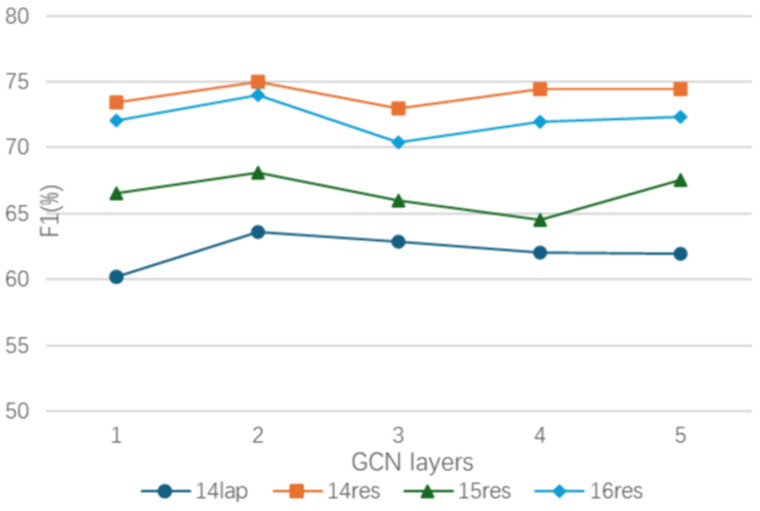
The experimental results of different number of GCN layers.

In order to explore the effect of the number of GCN layers on the performance of the method, experiments were conducted on the four datasets of ASTE-Data-V2. The number of GCN layers was increased from 1 to 5, and the F1 score was used as the evaluation metric. As demonstrated in Fig 7, it is evident that the maximum F1 score for each dataset is obtained when the number of GCN layers is configured to 2. When the number of GCN layers is set to 1, the feature extraction is inadequate, resulting in the failure to extract useful features. When the number of GCN layers exceeds 2 layers, it will cause overfitting problem, and it is also easy to introduce redundant information that will cause model performance degradation. Consequently, the number of GCN layers was selected 2.

### Case study

A case study was conducted to demonstrate the effectiveness of the proposed method. We selected seven examples from the ASTE-Data-V2 dataset for the purpose of comparing our method with the BDTF method. The seven sentences can be categorized into six distinct types based on failure types: sentiment polarity, comparative constructions, overlapping aspect terms, boundary identification, prepositional phrases, and long-range coordination. The results of the study are presented in Table 6.

**Table 6:** Case study.

Failure Type	Example	Golden Truth	BDTF	Ours
Sentiment polarity	The baterry is very longer.	(baterry,longer,positive)	(baterry,longer,negative)	(baterry,longer,positive)
	My laptop with Windows 7 crashed and I did not want Windows 8.	(Windows 7, crashed, negative) (Windows 8, not want, negative)	(Windows 7, crashed, neutral) (Windows 8, not want, negative)	(Windows 7, crashed, negative) (Windows 8, not want, negative)
Comparative constructions	The food was as creative as the decor and both worked.	(food,creative,positive) (decor,creative,positive)	(food,creative,positive) (decor,creative,positive)	(food,creative,positive)
Overlapping aspect terms	Our waiter was horrible; so rude and disinterested.	(waiter,rude,negative) (waiter,horrible,negative) (waiter, disinterested, negative)	(waiter,rude,negative) (waiter,horrible,negative) (waiter, disinterested, negative)	(waiter,rude,negative) (waiter,horrible,negative) (waiter, disinterested, negative)
Boundary identification	It has all the expected features and more +plus a wide screen and more than roomy keyboard.	(screen,wide,positive) (keyboard, roomy, positive) (features, expected, positive)	(screen,wide,positive) (keyboard,more than roomy,positive) (features,expected,positive)	(screen,wide,positive) (keyboard, roomy, positive) (features, expected, positive)
Prepositional phrases	A weakness is the chicken in the salads.	(chicken in the salads,weakness,negative)	(chicken, weakness, negative)	(chicken, weakness, negative) (salads, weakness, negative)
Long-range coordination	I reccomend the fried pork dumplings, the orange chicken/beef, and the fried rice.	(fried pork dumplings, reccomend, positive) (orange chicken/beef, reccomend, positive) (fried rice, reccomend, positive)	(fried pork dumplings, reccomend, positive) (orange chicken/beef, reccomend, positive)	(fried pork dumplings, reccomend, positive) (orange chicken/beef, reccomend, positive) (fried rice, reccomend, positive)

Two examples are provided to illustrate errors in sentiment polarity prediction. In the first instance, the opinion term is “longer” and the aspect term is “baterry” which should correspond to a “positive” sentiment polarity. However, the BDTF method incorrectly predicts the sentiment polarity as “negative.” In the second example, both our method and BDTF successfully extract two triplets. Nevertheless, BDTF method incorrectly predicts the sentiment polarity of the triplet (Windows 7, crashed, negative) as “neutral,” potentially due to its failure to adequately incorporate contextual semantic information.

Regarding the example containing a comparative construction, the sentence contains two triplets. While the BDTF method accurately predicts both triplets, our method identifies only one. Specifically, our method fails to predict the triplet (decor, creative, positive) due to its inability to recognize the comparative construction “as…as…”. This limitation may be attributed to insufficient semantic comprehension of this specific type of comparative construction.

In the case of overlapping aspect terms, both our method and BDTF accurately identify all triplets within the sentence, including the count of triplets, the recognition and pairing of aspect and opinion terms, and the prediction of sentiment polarity.

For the example concerning boundary identification, which contains three triplets. Both our method and BDTF successfully identify the number of triplets. However, our method achieves perfect accuracy across all triplets, whereas BDTF predicts one erroneous triplet due to the incorrectly opinion terms extraction. This result demonstrates the effectiveness of the boundary-aware contrastive learning module in our approach, confirming its capability to improve boundary detection.

The example involving prepositional phrase contains one triplet. BDTF predicts a single triplet, while our method predicts two triplets. However, all predicted triplets from both methods are incorrect. In this sentence, the aspect term should be “chicken in the salads.” However, the BDTF method identifies only “chicken” as the aspect term, while our approach erroneously extracts both “chicken” and “salads” as aspect terms. The model fails to recognize that “in the salads” is the attribute of “chicken,” leading to incorrect aspect term identification. The cause may lie in the model‘s limited ability to understand complex syntactic structures.

In the case involving long-range coordination, the sentence contains three triplets with overlapping opinion terms. Our method successfully extracts all triplets, but BDTF identifies only two triplets. The BDTF method fails to capture the aspect-opinion relationship following the conjunction “and” likely due to its limitations in parsing long-range coordination and its incomplete understanding of global syntactic dependencies.

In summary, the BDTF method demonstrates insufficient understanding of the semantic information of sentences, and fails to leverage the syntactic information of sentences and external knowledge to enhance the model‘s extraction of potential syntactic and semantic features of sentences. Compared with the BDTF, we use the external knowledge of syntactic dependency tree, part of speech and SenticNet to enhance the model‘s ability to understand sentences and extract triplets more accurately. However, through the above examples, it can be found that our model also has some limitations in dealing with some more complex syntactic structures. Future work should focus on incorporating deep syntactic analysis and fine-grained semantic reasoning to improve the robustness and performance of triplet extraction.

### Computational efficiency and resource analysis

We conducted experiments on the 14lap and 14res datasets of ASTE-Data-V2, and reported the training and inference times, parameter counts and GPU memory usage. The results are shown in the Table 7. The experiments were conducted on 4090-24G GPU. The training time refers to the time required to complete one training epoch, while the memory usage refers to the peak GPU memory usage during one training epoch.

**Table 7:** The comparison of training time,inference time, parameter counts and memory usage.

Model	14lap				14res			
	Training time	Inference time	Parameter count	Memory usage	Training time	Inference time	Parameter count	Memory usage
BDTF	38 seconds	4 seconds	162047238	8.82GB	46 seconds	6 seconds	162047238	8.97GB
Ours	66 seconds	10 seconds	190188282	8.23GB	86 seconds	15 seconds	190883466	8.37GB

Compared with BDTF method, our method has increased the training and inference times and parameter counts. This is attributed to the incorporation of external knowledge such as syntactic dependency tree, part of speech and SenticNet, along with the design of dual encoders and a boundary-aware contrastive learning module, which collectively contribute to a more complex model architecture. However, our method achieves a slight reduction in GPU memory usage relative to the BDTF method. In future work, we will prioritize improving computational efficiency and inference time of our method.

### Significance tests

To further validate the effectiveness of our proposed method, we conducted significance tests on the four datasets of ASTE-Data-V2. The experiments employed the F1 score as the evaluation metric, reporting the average F1 scores and their standard deviations obtained under five different random seeds. We selected BDTF and the PBLUN model based on BDTF as comparative models, and the specific experimental results are shown in Table 8.

**Table 8:** Experimental results of significance tests on ASTE-Data-V2.

Model	14lap	14res	15res	16res
BDTF	61.74	74.35	66.12	72.27
PBLUN	62.51	75.03	66.88	72.89
Ours	63.14 (0.48)	74.73(0.32)	68.09(0.2)	74.02(0.53)

The experimental results of BDTF and PBLUN are taken from published papers. The results in () are standard deviations.

Experimental results demonstrate that, compared to BDTF, our method achieves average F1 score improvements of 1.4%, 0.38%, 1.97%, and 1.75% on the four datasets, respectively. When compared to PBLUN, our method achieves improvements in average F1 score of 0.63%, 1.21%, and 1.13% on the 14lap, 15res and 16res datasets, respectively, while experiencing a slight decline of 0.3% on the 14res dataset. The standard deviations obtained across the four datasets are 0.48, 0.32, 0.20, and 0.53, respectively. The relatively large standard deviations observed on 14lap and 16res indicate more pronounced variability in the single-run experimental results on these two datasets, whereas the results on 14res and 15res remain relatively stable.

The results indicate that the proposed method demonstrates superior average performance, suggesting its enhanced capability in extracting the features of sentences effectively. However, significant fluctuations observed in two datasets may be attributed to the highly complex architecture of the model. Consequently, in subsequent research, we will consider implementing lightweight modifications to our proposed model to improve its stability.

## Conclusion

In this paper, we propose a novel end-to-end approach to extract sentiment triplets. Our proposed approach solves the problems of insufficient extraction of potential semantic and syntactic information in sentences, failure to take into account the information of the words themselves, and inaccurate identification of boundary tags in previous studies. Firstly, in order to fully exploit the semantic and syntactic information of the sentence and utilize the intrinsic information of the words themselves, syntactic dependency tree, POS information and SenticNet are introduced. In addition, two encoders are designed to adequately extract the potential features. Consequently, a feature fusion module is designed to facilitate the effective integration of these features. Finally, the inaccuracy of boundary tags recognition is addressed through the development of a boundary-aware contrastive learning module. This module enables the model to more accurately learn the boundary relationship between aspect and opinion terms, thereby improving the accuracy of boundary tags recognition. A series of experiments have been conducted on our proposed method, and the results demonstrate its effective performance.

However, our method also has some limitations. The effect of triplet extraction on special sentences needs to be further improved. Therefore, in future work we need to further integrate deep syntactic analysis and fine-grained semantic reasoning to further improve the performance of triplet extraction. In addition, the model‘s lightweight design also should be taken into consideration to improve computational efficiency while reducing resource consumption.

## Supporting information

S1 File Datasets. (ZIP)
